# Genetic Epidemiology of Hepatitis B Virus in Africa: A Review

**DOI:** 10.1016/j.gastha.2026.100968

**Published:** 2026-04-15

**Authors:** Samson Laribik Dujing, Eugene Dogkotegne Kuugbee, Williams Walana

**Affiliations:** 1 Department of Clinical Microbiology and Immunology, School of Medical Sciences, C.K. Tedam University of Technology and Applied Sciences, Novrongo, Ghana; 2 Department of Clinical Microbiology, School of Medicine, University for Development Studies, Tamale, Ghana

**Keywords:** HBV, Genotypes, Subgenotypes, Africa

## Abstract

**Background and Aims:**

Hepatitis B virus (HBV) is an enveloped and hepatotoxic virus belonging to the hepadnaviridae family, and it has the potential to cause both acute and chronic hepatitis. HBV remains a major public health threat, particularly in Asia, Africa, and South America, resulting in several deaths even though there is currently a safe vaccine against it.

**Methods:**

This systematic review examined the geographic distribution of HBV genotypes across Africa. We conducted a literature search on the major databases using key search strategies and a thorough combination of terms for HBV genotype distribution, from January 1, 2014, to January 31, 2025.

**Results:**

The findings revealed varying HBV genetic diversity in African regions, with some studies demonstrating similar or consistent results. Genotypes A, D, and E were commonly reported. However, genotype E was the most predominant, while genotype A was highly variable across the continent, with subgenotype A1 being the most common. Finally, some studies discovered novel recombinant strains from HBV genotypes and subgenotypes.

**Conclusion:**

There were limited reports on HBV genotype across Africa, with scarce genotype and clinical linkages, making HBV genotype epidemiology a relevant gap for informed clinical decision-making in hepatitis B treatment.

## Introduction

Hepatitis B virus (HBV) is an enveloped and hepatotoxic virus belonging to the hepadnaviridae family,^1^ and it has the potential to cause both acute and chronic hepatitis. HBV remains a significant public health threat, particularly in Asia, Africa, and Southern America, resulting in several deaths even though there is currently a safe vaccine against it.^2^ Chronic hepatitis B may develop into liver cirrhosis within 5 years in about 8%–20% of infected individuals,^3^ with over 350 million people chronically infected globally.^4^ Chronic hepatitis B is a continuously changing process which differs in stages, showing the complex interaction between infection and immune response.^5^

The progression of hepatitis B infection is categorized into 5 distinct clinical phases: immune-tolerant, hepatitis B e antigen (HBeAg)-positive immune-active, immune-inactive, HBeAg negative immune-active, and hepatitis B surface antigen (HBsAg) loss or resolved HBV infection. The duration of these phases can vary, and patients may not necessarily experience them in a continuous or sequential manner.^6^

HBV is a heterogeneous virus with 10 genotypes ranging from A to I, with J being putative, which are currently known, but genotypes A and D are most ubiquitous worldwide.^7^ It is evident that various genotypes and subgenotypes demonstrate different geographic distributions and are associated with disease progression, clinical progression, response to antiviral treatment, and prognosis.^8^ Epidemiologically, it is important to track the patterns of HBV transmission, and characterizing HBV genotypes in populations could inform the transmission pattern. HBV sequence characterized by >8% nucleotide difference predicts genotype variations, and 4%–8% nucleotide differences suggest subgenotype differences.^2^

There are over 40 related subgenotypes associated with HBV genotypes that have been discovered so far. In Africa, HBV genotype A is the most common in central and southern Africa, E in western Africa, and D in northern Africa.^9^ The infection process, clinical symptoms, and response rate to HBV therapy have a strong correlation with the virus genotypes and subgenotypes.^10^

There is increasing evidence showing that HBV genotyping plays a significant role in determining the progression of HBV diseases and proposing the right antiviral therapy, even though some genotypes are related to specific types of prognoses, like acute forms of the disease. Accordingly, reports have shown that genotype A develops more rapidly in patients compared to genotype D, thereby presenting some treatment difficulties.^11^

In recent times, there have been different methods of HBV infection detection in the laboratory, which mainly encompass HBV serological tests, hepatitis B “two half” tests, liver fibrosis index tests (laminin, type III procollagen peptide, hyaluronic acid, type IV collagen, etc), and HBV identification using molecular techniques. For instance, HBV gene identification, HBV genotype identification, detection of HBV drug resistance, and so on. These laboratory tests collectively provide key information to our understanding of the dynamic of HBV in terms of diagnosis, treatment, monitoring, and evaluation.^12^

This review focused on providing an overview of the distribution of HBV genotypes in Africa. With emphasis on HBV genotypes’ influence on hepatitis B disease progression and as such the need to know the geographic prevalence of HBV genotypes and subgenotypes. This will help inform clinicians on the adoption of contemporary and practical strategies to control this public health menace.

## Methodology

### Search Strategy

Peer-reviewed articles which are based on HBV genotypes and subgenotypes distribution in Africa were reviewed using the systematic review format by employing the following methods: (1) forming the research question, (2) identifying relevant studies, (3) the criteria for including/excluding studies, (4) finding and reviewing the literature, and (5) selecting studies and critical assessment and their detailed description.^13^

We conducted a literature search on the following established databases: Medline/PubMed, SCOPUS/Science Direct, EBSCO, and Google Scholar using key search terms. The keywords search terms in the topic were combined and used to construct Boolean search strings which included “HBV genotypes” or “HBV genetic diversity” or “HBV molecular characterization” or “HBV sub-genotype” AND “HBV genotype prevalence” or “HBV sub-genotype prevalence” or “Africa” AND “Sub-Saharan Africa” or “West Africa” or “Tropical Africa” or “Sahel Africa,” in the search process to identify relevant literature. All studies in Africa were included. In order to obtain the most current literature, filters for publication were at least 10 years old. During the review process, we assessed the relevance of titles and abstracts of the articles under review. Mendeley Reference Manager was used to manage full-text articles of relevant studies that were retrieved. The articles that were used included articles published in peer-reviewed journals, cross-sectional studies, and cohort studies. We focused on reviewing studies that investigated HBV genotypes or subgenotypes distribution in hepatitis B reactive humans only. We adopted and adhered to reporting items using the guidelines for the Preferred Reporting Items for Systematic Review and Meta-Analysis (PRISMA).

### Study Selection

This review on HBV genotype distribution included articles published in journals from January 2015 to December 2024. At least 2 authors independently selected studies, extracted them based on their titles and abstracts, and assessed the risk of bias in the studies that met the inclusion criteria. All disagreements and discrepancies were discussed between all reviewers and resolved by the third author. Studies or articles written in English and focused on HBV genotype distribution were included. However, articles that did not have any of the key search terms and were not written in English were excluded. Also, duplicates were sorted out to minimize bias.

#### Participants

The population for this systematic review is individuals with hepatitis B infection in Africa. Participants should have been laboratory-diagnosed HBsAg positive. No restrictions are placed on participant age, gender, ethnicity, or other demographic characteristics.

#### Intervention

No intervention.

#### Comparator

No comparator.

#### Outcome

The primary outcome of this study is to identify HBV genotypes in Africa.

The selection process is represented in a PRISMA flow diagram in Figure.

**Figure:**
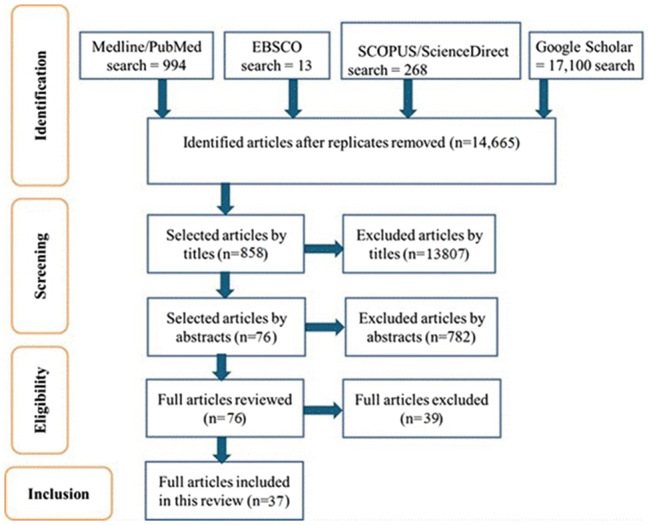
PRISMA flow diagram employed for the identification and screening of studies.

### Critical Assessment and Their Detailed Description

Articles that met the inclusion criteria and were deemed to have qualified for the study had the name of the author(s), year of publication, study objectives, study focus, study location, and major findings of the study extracted. The study findings were categorized into key themes, and gaps in the literature were identified for future research, and likely limitations of existing literature were addressed. This review has its findings presented in a narrative form.

## Results

### Overview of Studies on HBV Genotype Distribution Across Africa

Table presents a summary of 37 peer-reviewed articles included in the review, out of the overall total of 14,665, which were retrieved from Medline/PubMed, Scopus/Science Direct, Google Scholar, and EBSCO. This review only focused on articles or studies of interest conducted in African countries. The distribution of studies by countries was as follows: Ghana (4), Nigeria (5), DRC (5), Ethiopia (5), Gambia and Senegal (1), Zambia (1), Cape Verde (1), Eritrea (1), Cameroon (1), Burkina Faso (1), Niger (1), Kenya (1), South Africa (1), Egypt (1), Algeria (2), Gabon (2), Mozambique (1), Madagascar (1), and Botswana (2). The majority (33) of articles adopted were cross-sectional studies, and 1 study had a cohort design, another study was a clinical trial, 1 retrospective study, and the last study was a longitudinal study.

**Table:** Selected Articles on HBV Genotype Distribution in Africa

S/N	Author/Year	Study design	Study objectives	Focus of study	Study location	Major findings of study
1	Ambachew et al,^14^ 2018	Cross-sectional	To investigate genotypes, mutations, and virological characteristics of HBV isolates	Identify HBV genotypes/serotypes	Hawassa, southern Ethiopia	1. Among HBV genotype A donors, approximately 45.5% (27 of 58) had viral loads near 4.3 log IU/mL, while only around 11% (3 of 27) showed levels above 4.3 log IU/mL; this difference was statistically significant (*P* = .002) 2. Although 13 out of 58 genotype A subjects exhibited HBeAg reactivity compared to only 2 out of 27 genotype D donors, this difference did not prove statistically significant 3. In the sequencing of 85 isolates, 68.2% (58 isolates) were identified as genotype A, with the remaining 31.8% (27 isolates) classified as genotype D. 4. Every genotype A isolate was assigned to the adw2 serotype, whereas genotype D isolates were divided among 3 serotypes: 21 were ayw2, 5 were adw2, and 1 was ayw3. 5. The occurrence of the adw2 serotype was significantly higher in HBV/A donors compared to HBV/D donors (*P* < .001).
2	Dongdem et al,^15^ 2016	Cross-sectional	To determine the distribution of HBV genotypes in CHB patients	Identify HBV genotypes	Accra, Ghana	1. Genotype A was identified in 8 cases (7.2%), genotype D in 3 cases (2.7%), while genotype E was the most frequently detected, appearing in 47 cases (42.3%). 2. The most prevalent genotype overall was E. Among male subjects, genotype E was observed in 21 cases (47.7%), followed by genotype A in 4 cases (9.1%). 3. A comparable trend was seen in females, where genotype E was the most commonly reported in 26 cases (38.8%), with genotype A following at 4 cases (6.0%). Genotype D was the least frequent in both sexes.
3	de Pina-Araujo et al,^16^ 2018	Cross-sectional	Investigating HBV characteristics in HBsAg-positive individuals	Identify HBV genotypes/subgenotypes	Sao-Vicente, Cape Verde	1. The study reported that HBV/A1 rate was 57% while HBV/A2 was 22%, followed by HBV/E at a rate of 20% and finally, HBV/D had 1% rate. A total 54 isolates were reported. 2. The HBV genotype together with the subgenotypes rates was found to vary in the islands. 3. Majority of the isolates (81%) were HBV/A1, while the remaining were HBV/A2 isolates, and common in Europe. 4. The HBV/A1 was similar to South American sequences into the Asian-America clade that may be due to the spread of common African ancestors via slave trade.
4	Wangui et al,^17^ 2024	Cross-sectional	Determine the rates of HBV genotypes among patients	Identify HBV genotypes	Moi, Kenya	1. Genotype A was the most prevalent at 34 (79.1%), B was 5 (11.6%), C and D was 0 while E–J was 9 (20.9%). 2. Also, all cases of genotype B were related with coinfection of genotype A. 3. The commonest genotype was A, followed by mixed coinfection of genotype A/B.
5	Maryse et al,^18^ 2021	Cross-sectional	The characteristics of HBV isolates from patients and advanced grouping of subgenotypes A.	Identify HBV genotypes/subgenotypes.	West Africa, Gambia, and Senegal	1. The HBV genotype A sequences isolates presented different popular and agreed subgenotypes A (A1, A2, A6). 2. There were 4 isolates discovered grouped differently from the other subgenotypes. 3. Three were confirmed as an expanding clade of subgenotype A4, showing inter-subgenotypic nucleotide divergence higher than 4% compared to the other subgenotypes except with subgenotype A4 isolates (3.9%). 4. The last one from Senegal appeared to be an expanding subgenotype similar to the new clade of A4.
6	Anabire et al,^19^ 2023	Cross-sectional	To characterize the HBV genotypes in pregnant women.	Identify HBV genotypes	Northern Ghana	1. There were 3 HBV genotypes (A, D, and E) identified in the pregnant women. 2. The majority of 175 (91.6%) had genotype E, 9 (4.7%) had mixed genotype A and E, 5 (2.6%) had mixed genotype D and E, and 2 (1.1%) had mixed genotypes, A, D, and E. 3. A percentage of women with the varying HBV genotypes were not dependent on age.
7	Tufon et al,^20^ 2016	Cross-sectional	Determine the predominant genotypes and viremia levels in CHB.	Identify HBV genotypes	Cameroon	1. The mainly identified genotypes in order of predominance were A (47.4%), E (39.5%), C/E (3.9%), A/C (2.6%), A/E (2.6%), B (1.3%), A/B (1.3%), and B/C (1.3%). 2. The genotype E was greatly associated with higher mean viral load and mean Aspartate Aminotransferase (AST) levels.
8	Hamida et al,^21^ 2021	Cross-sectional	To investigate the genotype of HBV in CHB.	Identify HBV genotypes	Asmara, Eritrea	1. The most prevalent HBV genotypes found was D (21.3%), followed by C (17.2%), E (15.6%), with mixed genotypes and their rates at C/D (13.1%) and C/E (10.7%). 2. Also, the other genotypes and mixed genotypes were C/D/E (highest), A/D (high), followed by D/E, A (2.5%), and B, A/E, B/E, and A/D/C (least) were reported.
9	Nsokolo et al,^22^ 2018	Cross-sectional	Assess liver pathology-associated biomarkers in adults with HBV.	Identify HBV genotypes	Lusaka, Zambia	1. The study results showed that genotype A and E were evenly distributed.
10	Ampah et al,^23^ 2016	Retrospective study	To investigate the rates and genomic diversity of HBV.	Identify HBV genotypes/serotypes	Offin River Valley, Ghana	1. The results showed that all 52 typable samples belong to genotype E. 2. Which were found to all belonged to serotype ayw4.
11	Chiladi et al,^24^ 2023	Cross-sectional	Detect the predominant genotypes of HBV.	Identify HBV genotypes	Yenagoa, Nigeria	1. HBV/E and HBV/B were the most detected genotypes with at the rates of 82.4% and 11.8%, respectively. 2. HBV/B + E mixed infections were at the rates of 5.9%, observed in 2 female subjects within age group 26–35.
12	Kabamba et al,^25^ 2021	Cross-sectional	Determine seroprevalence and molecular characterization of HBV in blood donors.	Identify HBV genotypes and subgenotypes	Lubumbashi, DRC	1. The genotypes that were detected according to prevalence were E (53.1%), A (41.8%), A3/E (3.8%), and A1/E (1.3%).
13	Sanou et al,^26^ 2018	Cross-sectional	Investigate seroprevalence of HBV and HDV and their genetic diversity.	Identify HBV genotypes	Western, Burkina Faso	1. The main strains of HBV detected belonged to genotype E.
14	Maindo et al,^27^ 2019	Cross-sectional	Investigating the genetic diversity of CHB infection in donors.	Identify HBV genotypes and subgenotypes	Kinshasa, DRC	1. Analysis of HBV nucleotide sequences revealed that the majority of strains, 66.7% (10 out of 15), belonged to genotype A. This was followed by genotype E, comprising 26.6% (4 out of 15), and genotype D, which was the least frequent at 6.7% (1 out of 15). 2. Genotype A strains were further classified into subgenotype A1, quasi-subgenotype A3, and subgenotype A4, with quasi-subgenotype A3 being the most dominant among them.
15	Brah et al,^28^ 2016	Cross-sectional	The genetic characterization of HBsAg in CHB	Identify HBV genotypes/recombinant	Niamey, Niger	1. The prevalent HBV genotype discovered was genotype E (HBV-E) in Niger. 2. They discovered 2 recombinant forms including HBV-E/D and HBV-A3/E reported previously in blood donors in Niger and Ghana.
16	Chambal et al,^29^ 2017	Cross-sectional	To determine the predominance, genetic diversity, and HBV profile.	Identify HBV genotype	Maputo, Mozambique	1. Genotype A was the most prevalent, it was identified in 25/27 (92.6%) patients, while genotype E was present in 2/27 (7.4%) patients.
17.	Angounda et al,^30^ 2016	Cohort study	To determine the molecular characterization of HBV in CHB patients.	Identify HBV genotypes, subgenotypes and mutations.	Pointe Noire, DRC	1. Among the identified cases, 58 (70.7%) were classified as HBV genotype E, while 24 (29.3%) belonged to genotype A, which was further divided into 3 subgenotypes: A3 (66.7%), A4 (20.8%), and A6 (12.5%). 2. The prevalence of genotype A was notably higher among patients with chronic active hepatitis (CAH) at 33.3% and hepatocellular carcinoma (HCC) at 31.6%, compared to other groups.
18.	Matlou et al,^31^ 2019	Cross-sectional	To investigate the molecular characteristics of a rare HBV subgenotype D4 isolate.	Identify HBV genotypes/recombinant	South Africa	1. The primary isolate and cloned sequences formed a monophyletic cluster away from subgenotype D4 reference 2. Strains. 3. Further recombination analysis revealed that isolate ZADGM6964 was in fact a D4/E recombinant strain.
19.	Pennap et al,^32^ 2019	Cross-sectional	To investigate the genetic characteristics of HBV in CHB.	Identify HBV genotypes	Central Nigeria	1. The study reported that genotype E was the most prevalent at a rate of 44%, followed by genotype B at a rate of 34.5% while the least was genotype A at a rate of 13.8%. 2. Also, genotypes B and E mixed infection was at a rate of (6.9%), and being the first study that detected genotype B as a circulating variant in the country.
20	Makiala-Mandanda et al,^33^ 2017	Cross-sectional	To investigate the predominance and diversity of HBV infection in suspected cases of yellow fever.	Identify HBV genotypes	DRC	1. The HBV genotypes detected in the study included A, E, and D. 2. Among the 48 HBV DNA -positive samples, genotype E was the most prevalent, identified in 31 cases, followed by genotype A in 13 cases and genotype D in 4 cases.
21	Esmail et al,^34^ 2016	Cross-sectional	To determine the prevalence of HBV and identify genotypes	Identify HBV genotypes	Minia, Egypt	1. Among HBV DNA-positive samples, genotype B was the most prevalent, detected in 6 out of 12 cases (50%). 2. Genotypes C and D accounted for 33% and 16% of the samples, respectively. 3. Due to the limited number of genotypes analyzed, no distinct correlation between HBV genotype and viral load could be established.
22	Deressa et al,^35^ 2017	Cross-sectional	To investigate the rate of HBV and HIV genetic characteristics and therapeutic-resistant mutations in coinfected cases in a cohort of HIV patients.	Identify HBV genotypes	Northwest, Ethiopia	1. The majority of the subjects were infected with HBV genotype A.
23	Groc et al,^36^ 2019	Cross-sectional	To determine the rates and diversity of HBV and HDV.	Identify HBV genotypes and subgenotypes.	Gabon	1. The study showed the existence of 2 genotypes including HBV-E (n = 21) and HBV-A (n = 133). 2. Also, among the 133 HBV-A isolates, 126 clustered with subgenotype A3 and 7 with subgenotype A4.
24	Boyce et al,^37^ 2017	Cross-sectional	To investigate an HBV variant from Ghana whose initial partial sequence was identified as a recombinant.	Identify HBV genotypes/recombinant	Ghana	1. The study presents the full genome sequence of an HBV recombinant strain that combines elements from both genotype D and genotype E.
25	Shindano et al,^38^ 2017	Cross-sectional	To genotype HBV strains and to characterize the molecular type of HBV in South Kivu.	Identify HBV genotypes and subgenotypes	South Kivu, DRC	1. The study results showed that genotype A was the most predominant at a rate of 97.6% while genotype E was the least at a rate of 2.4%. 2. Most of genotype A strains were similar to subgenotype A1 strains while a few strains were linked with A2 subtype.
26	Gourari et al,^39^ 2019	Cross sectional	To characterize the genetic variation of hepatitis B virus (HBV) and hepatitis delta virus (HDV) strains present in Algeria while also assessing the prevalence of HDV infection through serological analysis.	Identify HBV genotypes, subgenotypes and recombinant	Algeria	1. Phylogenetic analysis of HBV strains showed the existence of genotypes D (86.5%) and A2 (11.76%). 2. The subgenotypes D are distributed as follows: HBV/D7 (43.5%), HBV/D3 (24.75%), HBV/D1 (16.8%), and HBV/D2 (14.85%). 3. A recombinant between genotypes A, E, and D was found.
27	Faneye et al,^40^ 2024	Cross-sectional	To identify the hepatitis B virus (HBV) genotype present in gastroenterology patients who test positive for hepatitis B surface antigen (HBsAg).	Identify HBV genotypes	Ibadan, Nigeria	1. Geno 1. Genotype A was the commonest at 15.7%, while genotypes B and E was the least with 1.2% rates each. 2. Genotypes A/C infection was prevalent among mixed infections at 40% rate, while genotype A/D had the least rates of mixed infection at 4.8%.
28	Shahen et al,^41^ 2021	Clinical trial	Investigate the genetic polymorphisms in the genes of adults prone to HBV infection.	Identify HBV genotypes	Algeria	1. The results showed that the distribution of genes between controls and infected subjects had little difference. 2. In the infected subjects, a rise in CC genotype was found at the same time with very low AC genotype distribution compared to the controls. 3. The AA genotype was found to be protective for HBV infection but the CC genotype had a higher HBV susceptibility.
29	Moges et al,^42^ 2022	Cross-sectional	To determine the genetic characteristics and HBV profile in pregnant women.	Identify HBV genotypes, subgenotypes, serotypes	Ethiopia	1. The 2 genotypes that were detected are A which was most prevalent (84.8%) and followed by genotype D 7 (15.2%). 2. The genotype A isolates were classified into subtype A1 and adw2 serotype. 3. Genotype D isolates were classified into 3 subtypes: 2 (4.3%) D2, 1 (2.2%) D4, and 4 (8.7%) D10. 4. Also, detected were ayw2 (10.9%), and ayw3 (4.3%) serotypes.
30	Meier-stephenson et al,^43^ 2020	Cross-sectional	Investigate the genetic characteristics and prevalence of HBV in a cohort of pregnant women	Identify HBV genotypes	Gondar, Ethiopia	1. The most prevalent HBV genotype among OHB individuals was D (97.3%).
31	Abe et al,^44^ 2023	Longitudinal study	To reveal the current status of hepatitis B and C virus (HBV and HCV) infections and the genetic diversity of the viruses.	Identify HBV genotypes/subgenotypes	Gabon	1. The subgenotype A1 was detected for the first time. 2. In addition to the previously identified HBV-A3, HBV-A5, and HBV-E strains.
32	Belyhun et al,^45^ 2022	Cross-sectional	To investigate HBV viremia among HBV and HIV dual and HBV-only-infected individuals.	Identify HBV genotypes/subgenotypes	Northwest, Ethiopia	1. HBV genotype A was the most commonly detected, accounting for 61.1% of cases, followed by genotype D at 38.3%, while genotype E was the least prevalent at 0.6%. 2. Among genotype A isolates, subtype A1 was the most frequently observed, making up 99.1% of cases, with A9 representing only 0.9%. In contrast, genotype D exhibited a broader range of subtypes, with D2 being the most prevalent (63.8%), followed by D4 (21.7%), D1 (8.7%), D3 (4.3%), and D10 (1.4%).
33	Andriamandimby et al,^46^ 2018	Cross-sectional	To analyze the genetic variability of hepatitis B virus (HBV) strains across various geographical regions.	Identify HBV genotypes	Madagascar	1. The most prevalent genotype/subgenotypes was E.
34	Uche et al,^47^ 2022	Cross-sectional	To investigate the genotypes of HBV among blood-borne infection patients.	Identify HBV genotypes	Lagos, Nigeria	1. Genotype A was the most predominant at a rate of (46.6%), followed closely by B (44.7%), E (23.8%), D (20.9%), and C (11.2%) being the least detected.
35	Mbamalu et al,^48^ 2021	Cross-sectional	To identify (HBV) genotypes and relevant mutations in the HBV DNA, among patients with chronic infection	Identify HBV genotypes, subgenotypes	South-Eastern, Nigeria	1. Genotyping show the detection of HBV genotype/subgenotype A1 (87.5%) and D (12.5%) in the participants
36	Mbangiwa et al,^49^ 2020	Cross-sectional	To investigate the HBV subgenotypes and viremia in blood donors.	Identify HBV subgenotypes	Botswana	1. The prevalent circulating subgenotypes were A1, and adw2 was the highest serotype at a rate of (36.1%), followed by D2 serotype ayw2 at (2.9%) and D3 serotypes ayw
37	Anderson et al,^50^ 2015	Cross-sectional	To identify circulating genotypes and to describe the mutations found in the HBV strains.	Identify HBV genotypes	Botswana	1. The most prevalent genotype was A found in 56 (80%) participants, D in 13 (18.6%), and 1 (1.4%) was genotype E.

AST, aspartate aminotransferase; CHB, chronic hepatitis B.

### Spectrum of Results

Eighteen of the studies were focused on the identification of HBV genotypes, 4 studies showed HBV genotype/subgenotype recombinant strains,^28^^,^^31^ while 11 studies were designed to investigate HBV genotypes and subgenotypes,^3^^,^^16^^,^^18^^,^^27^^,^^30^ and 4 studies reported on HBV genotypes and serotypes.

### Objective-Based Analysis of the Results

#### Hepatitis B virus genotypes

As summarized in Table, all the studies conducted in West Africa revealed the identification of 3 HBV genotypes A, D, and E, with the latter being the most predominant.^15^^,^^18^^,^^19^^,^^24^^,^^26^^,^^28^^,^^32^ Except for 3 studies, which reported genotype A as most prevalent in West Africa,^40^ with 1 study in Nigeria reporting for the first time a newly discovered HBV strain, HBV-B genotype.^32^ Conversely, 2 studies in East Africa reported HBV genotype A as the most prevalent,^14^^,^^17^ while 2 studies reported genotype D and genotype A as the most common genotypes.^21^^,^^29^

In Central Africa, most of the studies reported genotypes identified as A, E, and D, with HBV-E and HBV-A sharing dominance.^20^^,^^25^^,^^27^^,^^30^ For the 4 studies in Southern Africa, the distribution of HBV genotypes A, D, and E was reported by 2 studies, while the other reported on the D4/E.^22^^,^^31^ Also, in North Africa, the 3 studies identified reported varying results on HBV genotype prevalence.^34^^,^^39^^,^^41^

#### HBV subgenotypes and serotypes

As indicated in Table, 3 subgenotypes of genotype A were revealed as A3, A4, and A6,^30^ while another study showed the classification of genotype A strain into A1, A3, and A4, with A3 being the most predominant.^27^ The molecular analysis of HBV genotype A sequences isolated from patients from the 2 countries (Senegal and Gambia) identified separate clusters from other known and confirmed subgenotypes A (A1, A2, and A6).^18^ A study by de Pina-Araujo and team^16^ showed that out of 54 isolates, HBV/A1 had the highest prevalence (57%), followed by HBV/A2 (22%), HBV/E (20%), and HBV/D the least prevalent (1%), while another study revealed subgenotype A1 as the commonly circulating genotype.^49^ Generally, HBV genotypes and subgenotypes were unequally distributed across the African continent. HBV/A1 isolates were predominant (81%) belonging to the African origin, with the remaining isolates being the HBV/A2. Additionally, all genotype A samples were classified under the adw2 serotype. In contrast, genotype D samples were distributed across 3 serotypes: ayw2 (21 out of 27 cases), adw2 (5 out of 27 cases), and ayw3 (1 out of 27 cases). The adw2 serotype was notably more prevalent among HBV/A donors compared to those with HBV/D.^14^

#### HBV mixed genotypes and recombinant strains

In the summarized articles presented in Table, 7 studies reported on mixed genotyping or coinfection, HBV/B + E mixed infections were seen with a prevalence of 5.9%, found among 2 female subjects within age group 26–35 (34), A3/E (3.8%), and A1/E (1.3%).^25^ The genotypes C/D/E (7.4%) A/D (4.9%), D/E (4.1%), A/E, B/E, and A/D/C (0.8%) were also present.^21^ Also, there were reports of genotypes C/E (3.9%), A/C (2.6%), A/E (2.6%), A/B (1.3%), and B/C (1.3%) mixed infection.^20^ Anabire and his team identified 9 (4.7%) had mixed genotypes A and E, 5 (2.6%) had mixed genotypes D and E, and 2 (1.1%) had mixed genotypes A, D, and E.^19^ However, in Kenya, C and D was 0 while E–J was 9 (20.9%).^17^ In Ibadan, Nigeria, the most prevalent genotype was A followed by mixed coinfection of genotype A/B. Genotypes A/C infection was the highest among mixed infections, with 40% prevalence, while genotypes A/D were the least prevalent mixed infection with 4.8%.^40^

However, there were 4 studies that reported on the HBV recombinant strains,^28^^,^^31^ and others identified 2 recombinant forms including HBV-E/D and HBV-A3/E, while further recombination analysis revealed that isolate ZADGM6964 was in fact a D4/E recombinant strain.^31^ The complete genome sequence of an HBV genotype D/E recombinant from Ghana is reported and compared with other recombinant strains discovered in Africa,^37^ and a recombinant between genotypes A, E, and D was found in Algeria.^39^

## Discussion

Three main areas of focus were discovered in this review: HBV genotyping identification, HBV subgenotyping identification, and the identification of recombinant strains and mixed genotypes. Understanding the genetic distribution of the HBV is very critical in providing information on disease progression and treatment strategies and provides improved clinical outcomes for hepatitis B-infected persons. All of the studies included were mainly in human populations with the majority of them being cross-sectional studies, one being a cohort study and one a clinical trial, highlighting the bird’s-eye view of the genetic diversity of the HBV in the African population.

This review incorporated studies that were based on investigating the various HBV genotypes and subgenotypes in hepatitis B-positive active persons. Dongdem et al in their study demonstrated some consistency with similar studies worldwide, revealing that genotypes A, B, C, D, and E were identified in different geographical locations within the continent. These were associated with HBV DNA viral load, but the most predominant genotype was E in West Africa,^15^^,^^19^ Table. Also, genotypes play a significant role in determining the molecular evolution and spread pattern of HBV. Some studies have demonstrated that clinical outcomes may be influenced by the variation in disease progression between genotypes. HBV genotypes vary according to the course of disease, developing mutations, and response to antiviral therapy. Hence, genotype identification is critical in determining patients who are at risk of disease progression and optimizing treatment.^37^

The findings of the review reported the discovery of some novel recombinant strains.^28^^,^^37^ The coinfection of the same host by different HBV genotypes may lead to the formation of recombinant viruses. Recombination of genotypes often happens in regions where several genotypes cocirculate and enhance diversification within individuals and in the general population, thus placing importance on HBV genetic variability with possible clinical implications.^37^^,^^39^

The findings of the study also suggest the reports of HBV genotype coinfection among patients with mixed genotypes in Moi, Kenya, having genotype A/B as the most predominant.^17^ Other studies reported that the identification of some mixed genotypes such as C/D/E, A/D, D/E, A, and B, A/E, B/E, and A/D/C.^20^^,^^21^ This aligns with a study conducted by Faneye and others,^51^ who attributed their results to having the highest prevalence of mixed genotypes within the age range of 25–34, which is most likely to be due to the transmission through high sexual activity and occupational exposure such as hairdressing, barbering, and other jobs that require the use of sharp objects like razors, clippers, and needles.^40^

Furthermore, it was revealed in the findings of the studies that sought to investigate HBV subgenotypes that genotype A was mainly investigated for its subgenotype (A1, A2, A3, A4, A6).^16^^,^^18^ This agreed with the results reported by Shindano et al who revealed HBV subgenotype A1 as being the most predominant among the genotype A subtypes.^38^

The HBV genotype variation within the continent may be attributed to method sensitivity and specificity, mutations, migration, sample quality, and lack of financial resources to increase the coverage of the study area and sites to cover a wider proportion of the study region. Furthermore, the majority of studies lack relations with clinical data, for instance, viral load, disease progression, and treatment responses, making it very difficult to fully evaluate the association between viral genetics and clinical phenotypes.

### Limitations

There is the likelihood of publication bias since the study included only articles written in English, excluding those written in other languages that could qualify to provide invaluable contribution or information to the study. This could influence our understanding of the accurate picture of the geographical distribution of HBV genotypes.

## Conclusion and Future Directions

This review explored the distribution of HBV genotypes across Africa. Genotypes A, D, and E were commonly reported, with genotype E as the most predominant, while genotype A exhibits regional specificity, with subgenotypes. Generally, HBV genotype reported in countries across Africa was relatively low, with majority as retrospective studies. There was a lack of connection between HBV genotypes and disease progression, severity, and treatment outcomes. This suggests the need for expanded longitudinal investigations into the genotypic epidemiology of HBV in the continent. Particularly, linking HBV genotype characteristics to diverse disease dynamics such as severity, disease progression, and treatment outcomes could be of clinical relevance. The identification of mixed genotype coinfection and recombinant strains of HBV justifies the need for continuous surveillance and the incorporation of HBV genotyping in clinical care to further inform the disease dynamics and clinical decision-making.
